# Eco-hydrological modelling of channel network dynamics—part 2: application to metapopulation dynamics

**DOI:** 10.1098/rsos.220945

**Published:** 2022-11-30

**Authors:** Leonardo E. Bertassello, Nicola Durighetto, Gianluca Botter

**Affiliations:** ^1^ Department of Civil and Environmental Engineering and Earth Sciences, University of Notre Dame, Notre Dame, IN, USA; ^2^ Department of Civil, Environmental and Architectural Engineering, University of Padua, Padova, Italy; ^3^ Dipartimento di Ingegneria Civile, Edile, Ambientale e Architettura, Università degli Studi di Padova, Padova, Veneto, Italy

**Keywords:** network dynamics, temporary streams, metapopulation, stream ecology, stochastic modelling, ecohydrology

## Abstract

Temporal variations in the configuration of the flowing portion of stream networks are observed in the large majority of rivers worldwide. However, the ecological implications of river network expansions/retractions remain poorly understood, owing to the lack of computationally efficient modelling tools conceived for the long-term simulation of river network dynamics. Here, we couple a stochastic approach for the simulation of channel network expansion and retraction (described in a companion paper) with a dynamic version of a stochastic occupancy metapopulation model. The coupled eco-hydrological model is used to analyse the impact of pulsing river networks on species persistence under different hydroclimatic scenarios. Our results unveil the existence of a climate-dependent detrimental effect of network dynamics on species spread and persistence. This effect is enhanced by dry climates, where flashy expansions and retractions of the flowing channels induce metapopulation extinction. Survival probabilities are particularly reduced in settings where the spatial heterogeneity of network connectivity is pronounced. The analysis indicates that accounting for the temporal variability of the flowing river network and its connectivity is a fundamental prerequisite for analysing in-stream metapopulation dynamics.

## 1. Introduction

For decades, metapopulation theory has represented a key means to advance our basic and applied understanding of the importance of dispersal in epidemiology, community ecology, conservation and evolution [1,2]. In freshwater ecology, landscape connectivity and suitability represent two of the main determinants of species dispersal. Landscape connectivity is the by-product of the heterogeneity of the landscape structure and the ability of different organisms to move for exploiting the available resources. It is generally defined as the degree to which a given habitat facilitates movement of the organisms across the landscape matrix [3,4]. This matrix can be viewed as a mosaic of habitats and land uses, such that habitat quality varies across the landscape, setting the stage for source–sink population dynamics. These attributes, and their spatio-temporal dynamics, are known to influence the persistence of aquatic populations and communities across a variety of hydrologic systems [5–7]. Riverine systems are a prominent example of such dynamic habitats: owing to their seemingly fractal nature, they create key preferential pathways for the propagation of species, with important consequences for biodiversity and species persistence [8–10]. River connectivity was typically studied relying on static networks (e.g. networks derived from digital elevation models), which display a universal dendritic structure [11,12]). Ecological consequences of the spatial configuration in such networks are well studied in the literature, and encompass effects on species richness, beta-diversity and population sizes [13,14].

While the spatial dimension of stream networks has been extensively analysed, their temporal dynamics, implied by the ever-changing wetness condition in the surrounding landscape, has received less attention by the ecological community. Fluctuations in streamflows along the network are the primary form of environmental variability in riverine ecosystems [15], owing to their impact on key physical attributes of river reaches [16,17] and the ensuing community composition (e.g. via direct effects on mortality and recruitment [18]). However, discharge variations are often coupled to significant temporal changes in the wet fraction of a river network, because individual river segments can completely dry out when the local run-off falls below a critical threshold. These ephemeral or intermittent stream networks fluctuate between dry and wet conditions, and are associated with important ecological values [7,19]. The temporary nature of stream networks ensures dynamic conditions to species, potentially generating transient connectivity windows and ephemeral dispersal opportunities available only when the flowing network reaches its maximum extent, when even the most persistent disconnections along the network get closed [20–22]. Streams that periodically dry out, on the other hand, may temporarily reduce the number, size and connectivity of suitable habitat patches [15,23]. The long-term persistence of a metapopulation arguably reflects the interplay between the extent of habitat fluctuations and the dispersal ability of the focus species [8,24–26]. Therefore, characterizing the joint dynamics of a temporary stream network and its aquatic metapopulation requires the development of interdisciplinary approaches at the interface between ecology and hydrology, able to capture how hydroclimatic fluctuations are perceived by the organisms hosted by river networks.

While the ecological implications of spatio-temporal variability in habitat suitability and connectivity have been already analysed in the eco-hydrological literature [21,27–36], the key question concerning how population occupancy responds to dynamic habitat changes in temporary streams is still unanswered. Until now, addressing the above issue was unfeasible, in the absence of reliable models for the simulation of event-based fluctuations in the extent and configuration of flowing stream networks over ecologically relevant timescales (see e.g. [32]).

To answer this question, we leverage the hydrological model proposed in a companion paper [37], and we couple it with a dynamic version of the stochastic patch occupancy model [38–41] to explore the effect of stream expansion and retraction on the persistence of a metapopulation. In particular, we present in what follows a set of synthetic applications of a novel eco-hydrological model for the joint stochastic simulation of the dynamics of a temporary river network and a related in-stream metapopulation. We will first describe the dynamic stochastic patch occupancy model (§2.2), in which the structure of the ecological network changes through time in response to unsteady hydroclimatic conditions. The results of the metapopulation model and the comparison between dynamic and static network scenarios are presented in §3.2. The analysis unveils how wet stream dynamics are mirrored by key topological network metrics and metapopulation occupancy under different settings. A discussion of the relevance of our findings (§4), and a set of conclusions (§5) then close the paper.

## 2. Methods

In what follows, we describe the proposed dynamic metapopulation model, and we summarize the key features of the underlying dynamic stream network model. For more details about the physical and theoretical hydrological set-up, the reader is referred to our companion paper [37].

### 2.1. Dynamic stream network modelling

In our mathematical framework, a dynamic stream network is represented by a set of nodes with arbitrary spatial coordinates and a binary state *X*_*i*_(*t*) that could be either 0 (dry) or 1 (active). Each node (*i*) is representative of the hydrological conditions in a uniform stream reach of length Δ*l*_*i*_ containing the node *i*. Temporal changes in the spatial configuration of the active network are simulated by assigning the status of each node in the network during a sequence of time steps. The time variability of the status of each node can be summarized by its local persistency, *P*_*i*_, which represents the marginal probability of node *i* being active.

The simulation of active river network dynamics is achieved by exploiting two common features of observed stream network expansion and contraction: (i) the synchronicity between the temporal variations of active length *L*(*t*) and streamflow at the outlet *Q*(*t*) (i.e. the network expands when streamflow increases, and contracts when *Q*(*t*) decreases [42,43]) and (ii) the persistency-driven hierarchical activation mechanism proposed by Botter and co-workers [44,45], according to which the nodes are always activated from the most to the least persistent during network expansion, and deactivated in the reverse order during retraction. Note, however, that *P*_*i*_ may vary non-monotonically in space, thereby generating disconnections along the active network. Thanks to the hierarchical mechanism, the set of possible spatial configurations of the active network is uniquely identified by assigning the local persistency *P*_*i*_ to each node. This is done linking *P*_*i*_ to the underlying climate and morphological features of the catchment. The temporal variability of the extent of the active network is instead driven by the hydrological signal *Q*(*t*). In this application, synthetic time series of catchment discharge with desired statistical features are generated using the analytical model proposed by Botter *et al.* [46]. In the model, daily effective precipitation is described by a Poisson process with frequency *λ*, rainfall depths are exponentially distributed with mean *α*, and a linear storage–discharge relation generates exponential streamflow recessions with rate *k*. For more information about the hydrological set-up that allowed us to generate the full spatial and temporal dynamics of a temporary stream network, the reader is referred to our companion paper [37].

### 2.2. Dynamic metapopulation model

A dynamic stochastic patch occupancy model is employed to simulate the presence–absence of a focal aquatic species over a dynamic stream network. In this application, we focus on a prototypical anadromous fish species living along the stream network, for which swimming represents the predominant mobility means. While it has been shown how directional dispersal in river networks might affect emergent biodiversity patterns [10,47], here we neglect the potential effect of this process on metapopulation persistency, since the main focus of the paper is to assess how the temporal variations of the active portion of the network impact affect species survival (see below). The model follows the same logic as that developed in [36] and represents a dynamic version of the widely adopted stochastic patch occupancy static model [38–41]. The latter is a homogeneous first-order Markov chain in which the state of the metapopulation at each time depends only on the occupancy pattern of the metapopulation at the previous time step. A binary state variable, *w*_*i*_(*t*), defines the occupancy of a given node: if site *i* is occupied at time *t*, *w*_*i*_(*t*) is set to 1, otherwise it is set to 0. From an initial distribution of occupied patches, dynamics are modelled as a discrete-time Markov chain. At each time step, the model allows unoccupied patches to be colonized by surrounding occupied patches with a probability $\Phi_{C,i}(t)$ defined as

2.1 $$\Phi_{C,i}(t)=\Phi [w_{i}(t)=1|w_{i}(t-\Delta t)=0].$$

Similarly, species in occupied patches can go extinct with a probability $\Phi_{E,i}(t)$ defined as

2.2 $$\Phi_{E,i}(t)=\Phi [w_{i}(t)=0|w_{i}(t-\Delta t)=1].$$

Then, for each node in the network and each time step, the probabilities of colonization and extinction events ($\Phi_{C,i}$ and $\Phi_{E,i}$) depend on colonization and extinction rates with exponential survival probabilities

2.3 $$\Phi_{C,i}(t)=1-\exp(-C_{i}(t)\Delta t)$$

and

2.4 $$\Phi_{E,i}(t)=1-\exp(-E_{i}(t)\Delta t),$$

where Δ*t* is the simulation time step (in this case 1 day), while *C*_*i*_(*t*) and *E*_*i*_(*t*) are the colonization and extinction rates for the *i*-node at time *t*, respectively. The key novelty introduced in the proposed dynamic version of the model is to consider the terms *C*_*i*_(*t*) and *E*_*i*_(*t*) as dependent on the current hydrological status of the river network according to the following equations:

2.5 $$C_{i}(t)=c\sum_{i\neq j}\exp\left(-\frac{d_{ij}(t)}{\delta }\right)S_{j}(t)w_{j}(t-\Delta t)$$

and

2.6 $$E_{i}(t)=\frac{e}{S_{i}(t)},$$

where *δ* is the species dispersal distance, *c* is the species colonization rate, *e* is the species extinction rate and *d*_*ij*_(*t*) is the pairwise distance between the reaches *i* and *j*. As *d*_*ij*_(*t*) is finite only along the active river network, dispersal cannot occur between disconnected patches (i.e. all nodes in the path between *i* and *j* must be active for the dispersal to be possible).

The suitability of node *i*, *S*_*i*_, is assumed here as function of the stream bed area where the primary production takes place [48]. We estimated the stream bed area as the product between the length of stream associated with the node, *δl*_*i*_, and its width, *W*_*i*_, which is assumed to be constant and is computed following [49] as a function of the upstream contributing area. Therefore, the habitat suitability of the node is defined as *S*_*i*_ = *δl*_*i*_ *W*_*i*_ *X*_*i*_(*t*), where *X*_*i*_(*t*) is the status of the node at time *t* (*X*_*i*_ = 1 → wet, *X*_*i*_ = 0 → dry). To avoid numerical problems in the calculation of *E*_*i*_ when *C*_*i*_ = 0, when a node is dry $\Phi_{E,i}$ is set to 1. Therefore, the number of available sites changes in time due to the expansions and contractions of the stream network, as the suitability of all dry nodes is set to zero. Equation (2.5) captures the time variability in habitat structure embedded by the amount of suitable habitat, *S*_*i*_(*t*), and patch spatial configurations, *d*_*ij*_(*t*). Equation (2.6) shows how the local extinction rate on the *i*-patch is inversely proportional to the suitability of node *i* [50]). The presence of rescue effects [50,51] in the extinction rate (equation (2.4)) is here neglected, because our primary objective is to investigate how environmental stochasticity affects species dispersal and persistence. Temporal fluctuations of colonization, *C*_*i*_(*t*), and extinction, *E*_*i*_(*t*), rates define two specific traits of the focal species: the ability of dispersing along the network to colonize new nodes, and the chance to become extinct.

The advantage of the dynamic metapopulation approach is to explicitly account for the spatio-temporal dynamics influencing habitat availability and accessibility. The model requires two fundamental variables of metapopulation dynamics: habitat suitability, which is considered here as function of the stream bed area, and connectivity, which is estimated based on dispersal across the inter-patch distance (*d*_*ij*_). Here, both these attributes are temporally variable, driven by unsteady hydroclimatic forcing as discussed in [37]. In this study, metapopulation dynamics are analysed mostly in terms of length of occupancy, Ω(t), which identifies the temporal dynamics of the fraction of river network that is occupied by the focal species. Ω(t)=1 implies that all the nodes in the network are occupied by the considered species, while Ω(t)=0 indicates that the species is extinct in the whole stream network. The impact of the active network variability on species survival was also analysed by comparing the results of each dynamical simulation with those of a static version of the metapopulation model, which was run over a fixed stream network with a constant flowing length equal to the mean network length of the corresponding dynamical case.

### 2.3. Numerical set-up

Our analyses comprise nine different scenarios, which arise from the combination of three possible climates (namely *Dry*, *Intermediate* and *Wet*, characterized by the parameters reported in table 1) with three contrasting spatial configurations of local persistency (persistency as a random function of space (1), persistency proportional to the topographic wetness index (TWI) (2) or to the contributing area (3)), as described in [37]. These three spatial configurations correspond to general realistic scenarios in which the occurrence of local surface flow is determined in different proportions by upstream contributing area, local slope and/or geolithological heterogeneities, and result in active networks that are either always completely connected to the outlet (3), or show an increasing number of disconnections (2 and 1, respectively). For each hydroclimatic scenario, we performed a set of 50 Monte Carlo simulations of the coupled dynamics of active network and metapopulation. These simulations correspond to different realizations of the rainfall process, last 1 year each, and were simulated with a daily temporal resolution. For each realization of *Q*(*t*) and *X*_*i*_(*t*), species persistence was simulated using both the static and dynamic metapopulation models. A pool of more than 200 virtual species was generated by randomly assigning values to the parameters *δ*, *c* and *e* from predefined ranges. *δ* ranged from 1 to 10 times the nearest neighbour distance (NND), while *c* ∈ [10^−5^; 10^2^] m^−2^ d^−1^ and *e* ∈ [10^−3^; 10^3^] m^2^ d^−1^. In what follows, we first report the full results of the sensitivity analysis on species survival for a set of virtual species spanning the entire range of the colonization and extinction rates. We ran the static and dynamic metapopulation model for each combination of the parameters, performing a set of 50 Monte Carlo simulations each and computing the number of times the species survived until the end of the simulation (365 days). Then, we show the results for a representative virtual species characterized by *c* = 0.015 m^−2^ d^−1^, *e* = 20 m^2^ d^−1^ and *δ* = 4 NND. While the former analysis allowed us to explore which type of species may or may not survive under different hydroclimatic conditions, the latter enabled us to improve our understanding of the different temporal dynamics in species occupancy variability between the static and dynamic metapopulation model. As such, the combination of *c*, *e* and *δ* values of the focus species was specifically selected to highlight the different behaviour of the metapopulation model under different hydroclimatic conditions.

**Table 1.** Summary of the parameters used for the simulation of network dynamics in the different hydroclimatic scenarios. The recession rate *k* is constant across all scenarios and equal to 0.35 d^−1^. Only *α*, *λ*_*P*_, *E*_*p*_ and *k* are independent parameters.

climatic scenario		dry (*D*)	int. (*I*)	wet (*W*)	
mean daily rainfall depth	*α*	15.0	12.00	10.0	mm d^−1^
mean rainfall frequency	*λ*_*P*_	0.25	0.40	0.55	d^−1^
potential evapotranspiration	*E*_*p*_	3.0	2.50	2.0	mm d^−1^
mean total precipitation	*P*_*t*_	3.75	4.80	5.50	mm d^−1^
actual evapotranspiration	*E*_*a*_	2.29	2.16	1.82	mm d^−1^
effective rainfall frequency	*λ*	0.10	0.22	0.37	d^−1^
rain freq. to recession rate ratio	*λ*/*k*	0.65	1.47	2.45	d^−1^
mean network persistency	$\bar{P}$	0.20	0.50	0.74	—

## 3. Results

### 3.1. Sensitivity analysis

**Figure 1.**
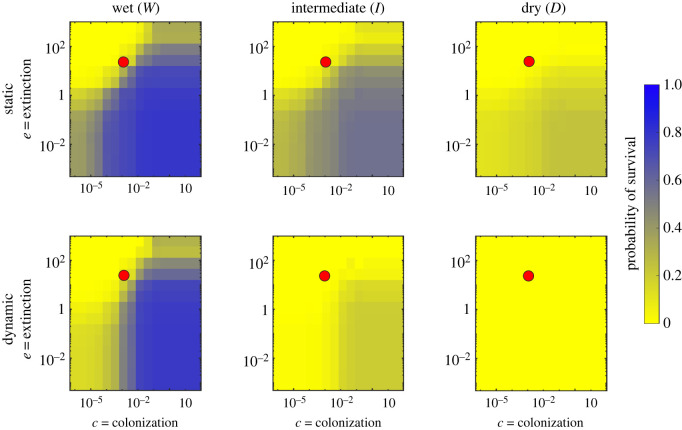
Comparison between static and dynamic version of the metapopulation model. These results refer to the case where the persistency is assigned as a random function of space.

**Figure 2.**
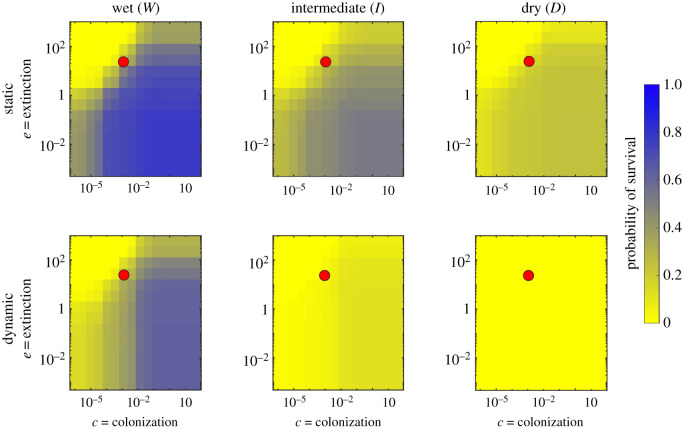
Comparison between static and dynamic version of the metapopulation model. These results refer to the case where the persistency is assigned based on the TWI.

**Figure 3.**
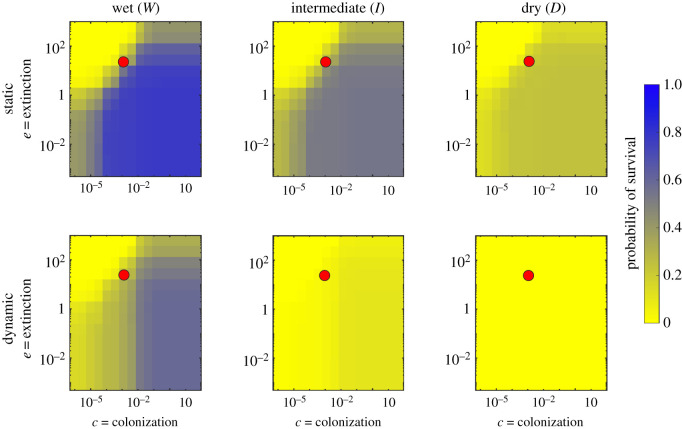
Comparison between static and dynamic version of the metapopulation model. These results refer to the case where the persistency is assigned based on the contributing area.

Patch occupancy dynamics are determined by patch attributes (suitability; gap distances) and species attributes (dispersal ability, colonization and extinction rates). While our companion paper [37] investigated how and why the river network is temporally variable in response to unsteady hydroclimatic forcing, here we present a sensitivity analysis on selected values of *e* and *c* to analyse the ecological implications of such variability. To disentangle the effects of such spatio-temporal variability, we compared the results of the proposed dynamic metapopulation model to its static counterpart. In particular, figures 1–3 show the results of this sensitivity analysis for the probability of metapopulation survival when the extinction and colonization constants vary in the range reported in §2.3.

Overall, the pseudocolour maps reported in figures 1–3 showed that, for a given hydroclimatic regime, the probability of survival tended to be similar across the different persistency scenarios, suggesting how metapopulation survival was primarily determined by the interaction between species traits and the hydroclimatic regime. During wet hydroclimatic regime, three main regions defined by the ratio *e*/*c* can be identified in the pseudocolour maps. For high values of *e*/*c* (top-left corner), the survival probability tended to zero, preventing metapopulation survival. Indeed, in this case the extinction process dominated and impeded species colonization even if the habitat might still have been suitable. On the other end, for low values of the *e*/*c* ratio (bottom-right corner) the probability of survival tended to one, mirroring a scenario where the given species were able to colonize the entire habitat, and thus survive. Finally, for intermediate *e*/*c* ratios (top-right and bottom-left corner) the individual values of *e* and *c* did play a role in defining the survival or extinction of the species. As the hydroclimatic conditions changed from wet to intermediate the different zonation tended to fade, with an overall decrease in the probability of survival which eventually collapsed to zero under dry hydroclimatic regimes, where the habitat suitability was too limited to promote successful colonization.

Another important result shown by the plots in figures 1–3 is that for fixed values of *e* and *c*, the probability of metapopulation survival under dynamic conditions was always lower than that observed under steady conditions. Indeed, the dynamic version of the metapopulation model predicted a lower value of occupancy for all the investigated scenarios. This was particularly evident when we compared the two dry cases. Here, for certain combinations of the colonization and extinction rates we still observed some viable metapopulation in the static case, while the dynamic model went to extinction in all cases.

The results presented above show what subset of species might survive given the hydrological regime of the river network. However, they do not give information about the temporal occupancy dynamics at the local scale, which is investigated in the following section, where we assumed a given combination of the colonization and extinction parameters (see the red circle in figures 1–3).

### 3.2. Coupled dynamics of active stream network and metapopulation occupancy

**Figure 4.**
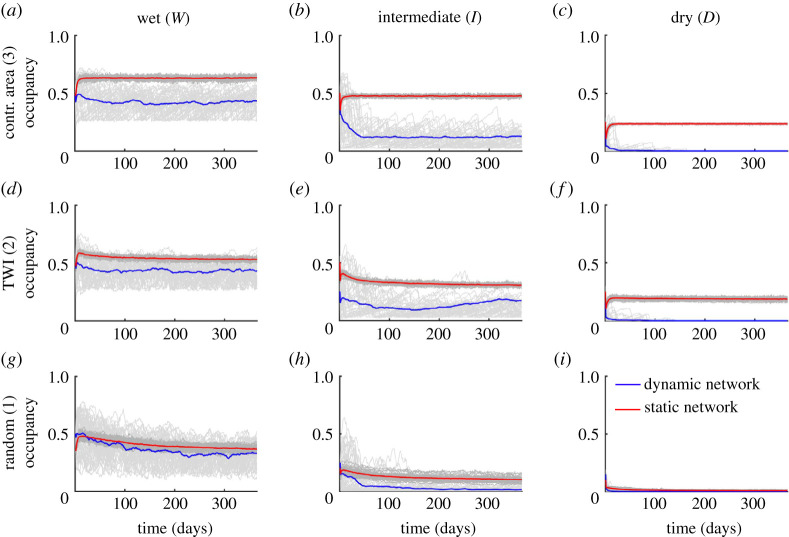
Comparison between the time series of river network occupancy computed using the dynamic metapopulation model and its static version. The grey lines refer to individual simulations (lighter grey for the dynamic model, darker grey for the static one), while the blue and red lines represent the average over 50 realizations. The comparison is repeated for each of the nine scenarios.

The time series of river network occupancy resulting from the application of the dynamic metapopulation model on the nine scenarios described above are shown in figure 4, which compares each dynamical simulation with a correspondent equivalent (i.e. average) static network. The 100 simulations (50 dynamic and 50 static) performed under each simulated scenario reflect the intra-annual variability of the underlying hydrologic conditions (grey lines). The blue and red lines, instead, show the average among the different Monte Carlo simulations performed with the dynamic and static metapopulation models, respectively. The temporal average of network occupancy was strongly affected by the variability of discharge and active length, with wetter climates leading to higher mean occupancy. This is a direct consequence of the fact that wet scenarios were associated with higher average active lengths. Therefore, at any given time a bigger portion of the network was available as habitat for the target species. Under wet conditions, the spatial pattern of local persistency did not seem to impact the average occupancy in the dynamic metapopulation model, as it only affected the spatial heterogeneity of the local occupancy. The random model, in particular, enhanced the presence of significant disconnections in the species persistency, originating patchy habitats along the network. For the intermediate and dry climates, instead, the networks corresponding to the random model (scenarios *I*1 and *D*1) had a lower mean occupancy than the others.

In all cases, the mean occupancy of the dynamic networks was lower than that of their static counterparts. This was particularly evident in the dry regimes, where the metapopulation survived only in static conditions, while the dynamic simulations all led to extinction. The lower mean occupancy was also accompanied by a higher temporal variability of the occupancy in all dynamic networks, as compared with the static scenarios. Accordingly, the coefficient of variation of network occupancy (average value of the temporal CV of Ω(t) during individual simulations) was enhanced by 4–6 times when the network dynamics were included in the model, increasing from 0.05 to 0.19 (from 0.07 to 0.45) in wet (intermediate) scenario. While the metapopulation survived in all the simulations of the wet regime (figure 4*a*,*d*,*g*), and in the contributing area and TWI scenarios of the intermediate cases (figure 4*b*,*e*), the majority of the simulations predicted metapopulation extinction for scenario *I*1, and metapopulation survival was null for the dry regime (figure 4*c*,*f*,*i*).

**Figure 5.**
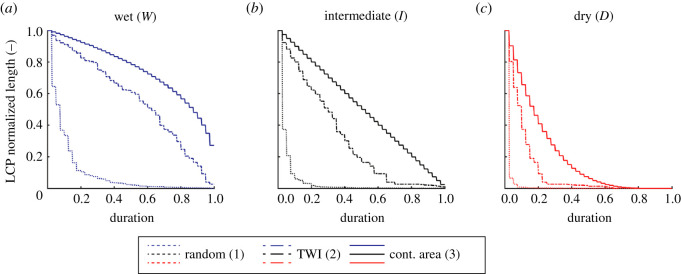
Duration curve of the length of the largest continuous portion of active network, LCP for wet, intermediate and dry hydrological conditions. The computation is repeated for the three different scenarios of local persistency: Contributing area, TWI and random, as described in §2.3.

The different occupancy dynamics observed under the nine scenarios analysed in figure 4 was explained by the underlying dynamics of the length of the largest continuous portion (LCP) of active network, which are summarized in figure 5. The latter figure shows the duration for which each possible value of LCP length is equalled or exceeded, and can be interpreted as a stream length duration curve [44] that only takes the connected active network into account. When local persistencies were assigned based on the contributing area, the ensuing networks were fully connected, and the LCP corresponds to the whole active stream network at a given time. As a consequence, the mean LCP length was equal to the mean network length, which also corresponded to the network length of the static scenario. This allowed the virtual species to get access to all the active reaches of the river network, thereby increasing the average network occupancy as compared with the other scenarios (for a given hydroclimatic setting). In scenarios *W*2*, I*2*, D*2 (with local persistency assigned by TWI), instead, a number of disconnections arose along the network, and several small active branches were disconnected from the outlet. However, in spite of the presence of these disconnections and the ensuing lower values of the LCP length, the mean LCP was only slightly smaller than the mean active length of the network. Consequently, the few disconnections only weakly hindered species dispersal, as compared with the previous case. Interestingly, in scenarios *W*1*, I*1*, D*1, when the local persistencies were spatially uncorrelated, significant sizes of the LCP were observed for very short durations even in the case of wet hydroclimatic conditions. In this case, the mean LCP was much smaller than the mean network length and, consequently, metapopulation dispersal was significantly hindered by the fragmentation of the active network. As climate varied from wet to dry, the curves in figure 5 moved to the left, suggesting that under dry conditions the size of habitat patches was relatively large only for the most expanded network configurations, a condition which seriously threatened metapopulation survival.

**Figure 6.**
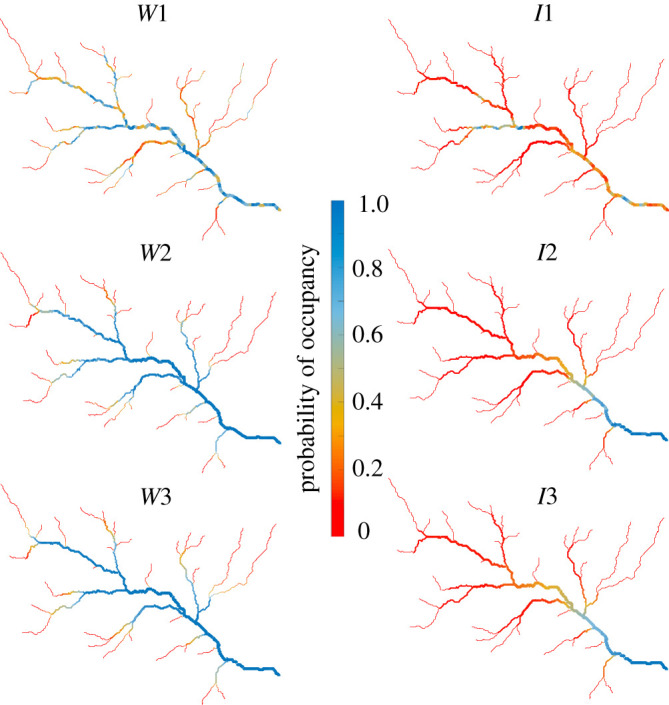
Maps of the probability of local occupancy (i.e. the fraction of time during which a node is occupied by the metapopulation) for six out of the nine simulated scenarios (wet and intermediate conditions).

The different hydrological dynamics experienced by the river network also affected the chances of metapopulation survival along the network, as shown in figure 6, which represents the spatial patterns of species occupancy at the node level for intermediate and wet conditions resulting from the dynamic metapopulation model. During the dry scenario (not shown), the focus species could not survive, and systematically went to extinction regardless of the spatial correlation of *P*_*i*_. The observed patterns of metapopulation occupancy only in part resembled the local persistency maps. The headwaters represented the portions of the river network with, on average, the lowest level of occupancy due to their highly intermittent dynamics and the more limited habitat suitability due to the reduced stream bed area. On the other hand, the main stream was characterized by the largest value of occupancy in all the scenarios, especially in the most downstream region of the stream network. Nevertheless, the random scenario (*W*1 and *I*1) represented the least favourable ones to maintain a viable metapopulation, due to the large number of disconnections that arose along the stream network.

## 4. Discussion

### 4.1. Eco-hydrological implications

The synthetic dynamic networks generated in the companion paper were used as domains for a network-scale ecological model devoted to the simulation of metapopulation dynamics in riverine environments. The application not only demonstrated the versatility of the proposed framework for eco-hydrological studies, but also provided novel insights on the role of network dynamics on species spread and survival in fluvial environments. Dynamic river networks, in fact, increase the temporal variability of network occupancy as compared with static streams. This is explained by the enhanced time variability of the portion of network that can be colonized by the target species: when the active network expands, additional nodes become available for colonization, and network occupancy generally increases. Conversely, when the network contracts, network occupancy is bound to shrink as more and more nodes dry out. However, a smaller mean network occupancy is observed in dynamic networks, with respect to the corresponding static streams with a constant, equivalent active length. This is related to the ephemeral nature of the nodes that are active when the network is relatively expanded, which makes them unsuitable for colonization, owing to the ecological lag between the node activation and the following node colonization. As such, the reduction of mean occupancy is more evident in dry climates, where only a limited number of nodes can sustain the metapopulation for prolonged time periods. These nodes act as starting points for species dispersal during the time periods in which the network is more expanded. The lower mean and the higher variability of network occupancy associated to dynamic networks is also reflected into a higher probability of extinction of the metapopulation in a dynamic setting, in accordance with previous studies [32].

Alterations to the spatial configuration of habitats and the temporal fluctuations in river flow may reduce the connectivity between temporary and permanent aquatic habitats and therefore could potentially limit their ecological use by aquatic organisms. Our analysis also revealed that enhanced stream disconnections appearing along the active network bear a significant signature on metapopulation dynamics, preventing species dispersal and promoting extinction. In dynamic streams, such disconnections may temporarily disappear during the network expansion, thereby creating ephemeral connectivity windows for species propagation along the network, with potentially relevant network-scale effect on metapopulation dynamics [21,29]. Although temporary, these hydrologically driven windows for connectivity may be efficiently exploited by fish [21] or amphibians [31,52], as demonstrated by modelling studies in which the contraction/expansion of water-related habitats was found to be a significant driver of species survival and reproduction. The key ecological implications of time-variable node connectivity pointed out in this study also suggest that future anthropogenic or natural alterations of current streamflow and active length regimes might severely impact species survival within river systems. For example, more frequent and severe droughts associated with climate change are likely to alter flow intermittence patterns, and arguably push temporary streams towards more ephemeral regimes [53], thereby generating less favourable conditions for the survival of metapopulations. Likewise, the observed increase in the number of dams [54] is likely to further exacerbate stream network fragmentation, with detrimental effects on species spread and persistence [19]. These dams can block critical fish migration routes between the river’s downstream floodplains and the headwaters, posing unnecessary risks to ecosystems and environmental services (e.g. biodiversity). Thus, protecting the hydrologic connectivity of these ephemeral ecosystems is fundamental to ensure species persistence, ecosystem integrity and river biodiversity. From this perspective, the spatially explicit analyses presented in this paper hint at the key impact of the number and position of stream disconnections for metapopulation persistency. Thus, the type of analysis proposed here can represent a useful tool for managers or conservation planners to make ecologically meaningful predictions of metapopulation survival in dynamic and disconnected freshwater ecosystems. One of the key advantages of the proposed approach relies on its ability to explicitly link the spatial configuration of stream disconnections along the network (as implied by e.g. ephemeral reaches, water abstractions, dams) and the corresponding network-scale ecological functioning of rivers.

### 4.2. Model limitations and future improvements

Our study showed the importance of coupling dynamic hydrological and ecological models to predict the patterns of species occupancy in dynamic stream networks. Here, the coupling was expressed based on habitat suitability, which linked the stream bed area that dynamically expands and contracts based on the hydroclimatic forcing to the probability of species colonization and extinction. This function was selected because the stream bed area is the zone where the primary production, which is an essential process at the basis of the food chain, takes place [48]. However, depending on the type of focal species, other attributes (e.g. temperature, water biochemistry) could be used to better define the habitat suitability.

Another potential limitation of our approach is represented by the assumption of unbiased dispersal, for which the direction of water velocity did not affect the movement of the target species. This assumption has been demonstrated to be valid for multiple fish species [55,56], but might be more problematic for other non-sessile organisms or vegetation species. Nevertheless, directional dispersal could be easily integrated in our metapopulation model by assigning weights to certain network links to favour either upstream or downstream movement. However, as biased dispersal interacts with network topology in complex ways, and may either promote or hamper metapopulation survival based on network geometry [57], characterizing its effect on dynamic stream networks is a very complicated task, which goes beyond the scope of the present paper.

Furthermore, the ecological model could be made more general by relaxing some of its assumptions (e.g. habitat suitability related to stream bed area, unbiased dispersal, no dispersal through dry nodes), thus allowing the description of various target species with different general traits. For example, for some species larger streams may not provide preferred habitat, requiring a different specification of the habitat suitability in our model. An additional improvement could be that of adding the possibility for the target species to survive on a given node for a certain period of time after the node had dried out, thereby allowing the model to describe species that can temporarily survive also in lentic habitats.

Finally, non-stationarity in species traits (*c*, *e*, *δ*) was also neglected in our analysis. This assumption is likely to be violated when evolution modifies species response to environmental variables. However, the timescale of the process is far beyond those covered in the proposed work.

## 5. Conclusion

In this paper, we coupled a stochastic approach for the simulation of channel network expansion and retraction with a dynamic ecological model to unveil the effects of spatio-temporal hydrological variability on metapopulation survival. Our simulations revealed the tight link between hydrological ecological processes taking place in temporary streams. The presence of river network dynamics was found to bear a detrimental impact on species spread and survival, owing to the fact that several portions of the network were available for colonization for a reduced amount of time. Hydrologic disconnections along the network, even when they were not permanent, significantly reduced the dispersal ability of the metapopulation, thereby enhancing the risk of extinction, especially when the number of disconnections was able to significantly alter the length of the LCP of active network (i.e. when the local persistency was poorly correlated in space).

As network dynamics were driven by climate, a strong climatic signature can also be found in the observed temporal patterns of species persistency. Temporary streams characterized by wet climates, in fact, were associated with higher but more stable degrees of occupancy of the target species. Conversely, the driest scenarios displayed more variable degrees of network occupancy and smaller survival probabilities. The metapopulation survival in these harsh hydroclimatic conditions is limited to those species who are characterized by smaller values of the *e*/*c* ratio, since they can quickly colonize those limited suitable habitats still present along the river network.

## Data Availability

All the data and code presented in this paper can be found in the Dryad dataset ‘Coupled stochastic modelling of hierarchical channel network dynamics and metapopulation persistency—Dataset’ available at the following link: https://doi.org/10.5061/dryad.0zpc86709 [58]. The data are provided in electronic supplementary material [59].
